# Optogenetic Manipulation of Postsynaptic cAMP Using a Novel Transgenic Mouse Line Enables Synaptic Plasticity and Enhances Depolarization Following Tetanic Stimulation in the Hippocampal Dentate Gyrus

**DOI:** 10.3389/fncir.2020.00024

**Published:** 2020-06-03

**Authors:** Thomas T. Luyben, Jayant Rai, Hang Li, John Georgiou, Ariel Avila, Mei Zhen, Graham L. Collingridge, Takashi Tominaga, Kenichi Okamoto

**Affiliations:** ^1^Lunenfeld-Tanenbaum Research Institute, Mount Sinai Hospital, Toronto, ON, Canada; ^2^Department of Molecular Genetics, Faculty of Medicine, University of Toronto, Toronto, ON, Canada; ^3^Basic Science Department, Faculty of Medicine, Universidad Católica de la Santísima Concepción (UCSC), Concepción, Chile; ^4^Department of Physiology, Faculty of Medicine, University of Toronto, Toronto, ON, Canada; ^5^Department of Cell and Systems Biology, Faculty of Medicine, University of Toronto, Toronto, ON, Canada; ^6^TANZ Centre for Research in Neurodegenerative Diseases (CRND), University of Toronto, Toronto, ON, Canada; ^7^Laboratory for Neural Circuit Systems, Institute of Neuroscience, Tokushima Bunri University, Sanuki, Japan

**Keywords:** cAMP, optogenetics, photoactivatable adenylyl cyclase (PAC), VSD imaging, electrophysiology, long-term potentiation, synaptic plasticity

## Abstract

cAMP is a positive regulator tightly involved in certain types of synaptic plasticity and related memory functions. However, its spatiotemporal roles at the synaptic and neural circuit levels remain elusive. Using a combination of a cAMP optogenetics approach and voltage-sensitive dye (VSD) imaging with electrophysiological recording, we define a novel capacity of postsynaptic cAMP in enabling dentate gyrus long-term potentiation (LTP) and depolarization in acutely prepared murine hippocampal slices. To manipulate cAMP levels at medial perforant path to granule neuron (MPP-DG) synapses by light, we generated transgenic (Tg) mice expressing photoactivatable adenylyl cyclase (PAC) in DG granule neurons. Using these Tg(CMV-Camk2a-RFP/bPAC)3Koka mice, we recorded field excitatory postsynaptic potentials (fEPSPs) from MPP-DG synapses and found that photoactivation of PAC during tetanic stimulation enabled synaptic potentiation that persisted for at least 30 min. This form of LTP was induced without the need for GABA receptor blockade that is typically required for inducing DG plasticity. The paired-pulse ratio (PPR) remained unchanged, indicating the cAMP-dependent LTP was likely postsynaptic. By employing fast fluorescent voltage-sensitive dye (VSD: di-4-ANEPPS) and fluorescence imaging, we found that photoactivation of the PAC actuator enhanced the intensity and extent of dentate gyrus depolarization triggered following tetanic stimulation. These results demonstrate that the elevation of cAMP in granule neurons is capable of rapidly enhancing synaptic strength and neuronal depolarization. The powerful actions of cAMP are consistent with this second messenger having a critical role in the regulation of synaptic function.

## Introduction

Hippocampal synapses play a critical role in learning and memory and exhibit post-synaptic NMDA receptor-dependent long-term potentiation (LTP) of synaptic transmission (Collingridge et al., 1983; Moser et al., 1998; Gilbert et al., 2001). The postsynaptic cAMP/PKA pathway at both the CA3-CA1 and medial perforant path to dentate gyrus (MPP-DG) synapses is known to be involved in protein synthesis-dependent LTP (late-phase LTP, or L-LTP or LTP2; Brandon et al., 1995; Nguyen and Kandel, 1996; Barad et al., 1998; Park et al., 2018). However, the mechanisms by how cAMP contributes to the induction of NMDA receptor-dependent LTP are still incompletely understood.

cAMP function in synaptic plasticity at excitatory synapses has conventionally been studied using pharmacological and genetic approaches (Abel et al., 1997; Barad et al., 1998; Wong et al., 1999; Navakkode et al., 2004; Govindarajan et al., 2011; Park et al., 2016). However, recent advances in optogenetic techniques have made it possible to control spatiotemporal signaling functions within living neurons (Kim et al., 2015; Murakoshi et al., 2017), thus providing powerful tools for addressing dynamic molecular processes at synapses. In this regard, optogenetic manipulation of cAMP production has begun to show promise. Endogenous photoactivatable adenylyl cyclase (PAC) has been reported from several species including *Euglena* and bacteria (Iseki et al., 2002; Stierl et al., 2011) which have been utilized to study cAMP functions (Jansen et al., 2015; Zhou et al., 2016). However, methods to control cAMP with spatiotemporal precision within intact brain tissue remain limited.

The DG is a gateway to the hippocampus where it plays a critical role in learning and memory (Moser et al., 1998; Gilbert et al., 2001). To examine the consequences of cAMP modulation in this pathway, we generated a PAC transgenic mouse line expressing a photoactivatable cAMP actuator in hippocampal DG granule neurons. We examined synaptic responses by electrophysiology and performed voltage-sensitive dye (VSD) imaging (Grinvald and Hildesheim, 2004; Homma et al., 2009; Peterka et al., 2011; Tominaga et al., 2013) to optically record activation of the entire DG at the circuit level under high temporal resolution (Chang and Jackson, 2006; Tominaga et al., 2018).

In acutely prepared hippocampal slices from PAC Tg mice, photostimulation of the cAMP actuator during MPP-DG pathway tetanic stimulation enabled potentiation of synaptic responses that lasted for at least 30 min. Furthermore, activation of PAC during the tetanus resulted in a greater depolarization within the dentate gyrus. The findings demonstrate that transiently enhanced cAMP signaling during MPP activity can potentiate synaptic transmission and facilitate the spread of neuronal depolarization. Importantly, these actions were manifest without any disinhibition, which suggests that neuromodulators acting *via* cAMP can negate the inhibitory influences on synaptic plasticity in the dentate gyrus.

## Materials and Methods

### Animal Care

Acute hippocampal slices were prepared following the guidelines of the animal use protocol approved by the animal care committees at The Centre for Phenogenomics (TCP; Toronto, ON, Canada) and Tokushima-Bunri University (Japan).

### Transgene Construction and Generation of PAC Mice

We generated a transgene containing the CMV enhancer-CaMKIIα promoter (1.3 kb), the coding region of RFP (tdTomato) fused with PAC which is codon-optimized for human expression (addgene ID 28134; Stierl et al., 2011) at the C-terminal and contains a polyadenylation signal. The resulting cDNA was subcloned into a custom plasmid vector (pMM403), including a gene for ampicillin resistance, for bacterial amplification. The 5.2 kb transgene was digested with SfiI +SalI to linearize the DNA and remove prokaryotic sequences. Tg(CMV-Camk2a-RFP/PAC)3Koka mice were generated by injecting the purified insert into the pronuclei of C57BL/6D2 mice at The Centre for Phenogenomics (TCP, Toronto, ON, Canada). Genotyping was performed by PCR to detect an 859 bp fragment using the following primers: 5′-TTCTCCGTTTGCACTCAGGAGC-3′ and 5′-GATGACGGCCATGTTGTTGT-3′. Founders were backcrossed with C57BL/6J mice for at least 10 generations. The official name for the resulting mouse line that we studied is C57BL/6J.B6 × B6D2-Tg(CMV-CamK2a-RFP/PAC)3koka.

### Electrophysiology

Acute hippocampal slices were prepared as previously described (Henderson et al., 2001) from adult (13–24 week-old) PAC transgenic mice and their wild-type (WT) littermates. After 1–2 h recovery, slices were transferred to a recording chamber perfused with an aCSF solution containing 124 mM NaCl, 3 mM KCl, 2.5 mM CaCl_2_, 1.3 mM MgSO_4_, 1.25 mM NaH_2_PO_4_, 26 mM NaHCO_3_, 10 mM glucose (pH 7.4, 30°C, 1.5 ml/min) equilibrated with 5% CO_2_/95% O_2_. Recordings of field excitatory postsynaptic potentials (fEPSPs) were conducted as previously described (Henderson et al., 2001) in the MPP-DG synapses without blocking inhibitory synaptic function. The stimulation electrode was positioned in the dorsal blade of the dentate molecular layer for MPP stimulation. Paired field responses were evoked by stimulating with an intensity (0.05 ms pulses, 40 ms apart) that yielded fEPSPs that were 40% of the maximum spike-free fEPSP size. Responses were evoked and acquired every 20 s throughout the experiment using an Axopatch 1D amplifier (Axon Instruments, San Jose, CA, USA) digitized at 20 kHz and measured by the slope (10–50% of fEPSP rising phase). The expressed PAC in DG granule cells was photoactivated using a blue LED light (1.5 mW, 5 min, EXFO, Canada) under an objective lens (4×, NA0.1, Nikon, Tokyo, Japan). Tetanic stimulation was induced with a bipolar matrix Pt;Ir microelectrode (FHC, Inc., CA, USA) using 100 Hz, 0.15 ms pulses delivered in four trains of 0.5 s duration, 20 s apart. In the case of photoactivation of PAC during MPP stimulation, the tetani were delivered at the end of the first minute of light exposure. In the time-course experiments, field responses were plotted by normalizing to the baseline fEPSP slope (average of the 10 min period before tetanus).

### Fluorescence Imaging of RFP-PAC in Hippocampal Slices

The hippocampal slices from PAC Tg mice were incubated with aCSF at room temperature in the microscope chamber. RFP fluorescence imaging of PAC was conducted using a confocal scanning microscope (Nikon C2 equipped with a 4× and 20× objective lenses, Nikon, Tokyo, Japan) with 543 nm (excitation laser) and 575–630 nm (emission). The fluorescence image was combined across the z-stacks composed of 30–50 sections taken at 5 μm intervals, and merged with the transmitted light image collected through a separate detector. The fluorescence images collected using the 20× objective lens were further deconvoluted (cellSens, Olympus, Tokyo, Japan).

### cAMP Measurement by ELISA

The hippocampal slices from PAC and WT littermate mice were homogenized in buffer (40 mM HEPES/Na, pH 8.0, 0.1 mM EGTA, 5 mM magnesium acetate, 1 mM DTT, and 0.01% Tween-20) by sonication, and centrifuged at 16,000 *g* for 15 min to clear large tissue debris. After isolation of the supernatant, the total protein concentration was measured using Bradford assay (protein assay kit, Bio-Rad, CA, USA) and adjusted the total protein concentration between PAC TG and WT for the photoactivation experiment. The lysate (100 μl) were excited for 10 min with a 455 nm LED (4.5 mW/mm^2^; ThorLabs, NJ, USA) on a plastic paraffin film (Parafilm M^®^, Bemis, USA) covered glass slide at room temperature, and cAMP was measured by ELISA (Enzo Life Sciences, NY, USA; Ryu et al., 2010; Stierl et al., 2011).

### Fluorescent Voltage-Sensitive Dye (VSD) Imaging

Fluorescent VSD imaging was carried as previously reported (Tominaga and Tominaga, 2016; Tominaga et al., 2019). Briefly, acute hippocampal slices (400 μm thick) were prepared from adult (10–21 week-old) PAC transgenic mice and maintained in aCSF, containing 124 mM NaCl, 2.5 mM KCl, 2 mM CaCl_2_, 2 mM MgSO_4_, 1.25 mM NaH_2_PO_4_, 26 mM NaHCO_3_, and 10 mM glucose, pH 7.4, gassed with 95% O_2_/5% CO_2_. After incubating for 1 h, each slice was stained for 15 min with 110 μl of VSD solution, containing 0.1 mM Di-4-ANEPPS (Molecular Probes, OR, USA) in 48.1% aCSF, 48.1% fetal bovine serum (Sigma–Aldrich, MO, USA), 2.5% ethanol, 1.17% distilled water, and 0.13% Cremophor EL (Sigma–Aldrich, MO, USA). After the washout of the dye with aCSF, slices were incubated for >1 h before the start of imaging.

Hippocampal slices (supported by Plexiglass ring) were placed in an immersion-type recording chamber (Tominaga et al., 2019). Slices were continuously perfused at a rate of 1 mL/min with aCSF at 31°C, gassed with 95% O_2_/5% CO_2_. The fluorescence VSD signals were recorded using a MiCAM Ultima imaging system with THT-01 epifluorescence optics (BrainVision, Inc., Tokyo, Japan), consisting of a two lenses system (Tominaga et al., 2000). A custom-made objective lens (Olympus MYCAM 5×/0.6 WI, Olympus, Tokyo, Japan) and a projection lens (PLANAPO, 1×, Leica Microsystems GmbH, Wetzlar, Germany). Excitation light was provided by a 150W halogen light source (MHF-G150LR, Moritex, Saitama, Japan) with an excitation filter (530 ± 10 nm) and an emission filter (>590 nm) for VSD imaging. VSD recordings of a duration of 400 ms (0.2 ms/frame, 2,048 frames) at 14 mW of excitation light were simultaneously obtained with electrophysiological recordings evoked with the delivery of a bipolar (0.2 ms pulse width) electrical stimulation to the MPP every 20 s. The intensity of the baseline electrical stimulation was determined to yield fEPSPs that were 40% of the maximum spike-free fEPSP size. Average of eight VSD recordings were presented as an optical signal. Recordings were taken before (baseline) and immediately following (time 0) tetanic stimulation (100 pulses × 100 Hz). PAC was photoactivated using 15 × 4 s duration pulses of excitation light (3.2 mW, 482/35 nm) every 10 s immediately before tetanic stimulation. The excitation wavelength used for PAC did not photobleach di-4-ANEPPS and vice versa. The imaging system provided a resolution of 18.2 × 18.2 μm at the objective plane (100 × 100 pixels resolution).

The fluorescence signal intensity before stimulation was averaged and used as the baseline reference intensity (*F*_0_). The change in fluorescence [Δ*F*_(t)_ = *F*_(t)_–*F*_0_] was normalized by *F*_0_ (Δ*F*/*F*_0_) and used as the optical signal. Optical signals were then Gaussian filtered in time and space by 5 × 5 × 3 (horizontal × vertical × temporal; IgorPro; WaveMetrics Inc., OR, USA). Pseudo-colored optical voltage maps were superimposed on the initial (pre-stimulation) gray-scale fluorescent image of the hippocampal DG area for a visual reference of the location. Since di-4-ANEPPS decreases in fluorescence when the membrane depolarizes, we chose to represent this change as a positive value to make the results more intuitive (Tominaga et al., 2000, 2002). The pseudo-colored 3D presentation of the VSD fluorescence changes were created using Origin Pro (OriginLab, Northampton, MA, USA). The threshold levels of the signal/noise were set independently of the peak fluorescence changes, using a set threshold of 40% of the maximum signal intensity at baseline to determine the distribution area of the fluorescence changes. For fitting the plots (Figure 5), we used OriginPro (OriginLab, Northampton, MA, USA).

**Figure 1:**
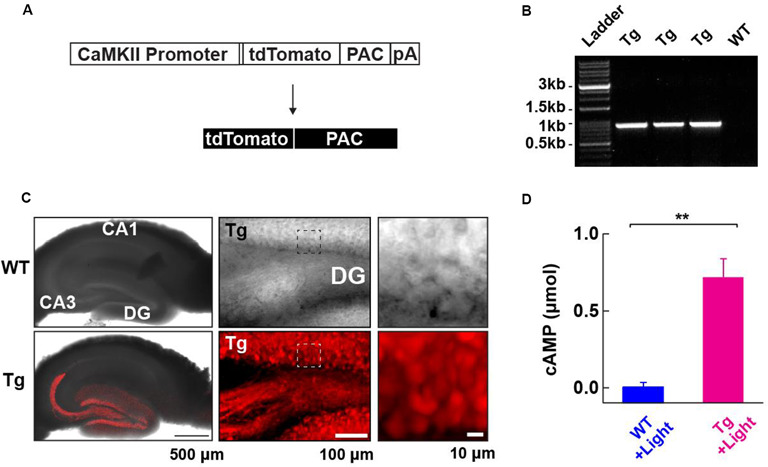
Generation and characterization of photoactivatable adenylyl cyclase (PAC) expressing transgenic (Tg) mice. **(A)** Schematic representation of the PAC transgene construct. **(B)** Genotyping of the Tg(CMV-Camk2a-RFP/PAC)3Koka mice. The specific PCR amplification of an 859 bp band for PAC in Tg, but not wild-type (WT) mice was detected on the agarose gel. **(C)** Distribution of RFP (tdTomato)-PAC in the hippocampal slice. Left upper: WT (wild type), Left lower: Tg (PAC transgenic mouse). The RFP fluorescence (red color) on the living hippocampal slice was imaged by a confocal microscope and merged with the transmitted light image. Note: the red labeling extending into the CA3 region reflects signals in the mossy fiber axons of the DG granule neurons. Middle: a transmitted light (upper) and the corresponding red fluorescence (lower) deconvoluted confocal images of the hippocampal DG area in a living PAC Tg mouse hippocampal slice. About 85% of DG granule cells (141/163 cells) showed red fluorescence signals above the background level. Right: The transmitted light (upper) and the corresponding red fluorescence (lower) images within the boxed region in the middle images. **(D)** ELISA-based detection of the light-dependent cAMP production in the WT and Tg hippocampal lysate (per mg total protein) after illumination (455 nm LED, 4.5 mW/mm^2^, 30 s; each *n* = 3). **p < 0.01 (unpaired *t*-test). Data are presented as mean ± SEM.

**Figure 2:**
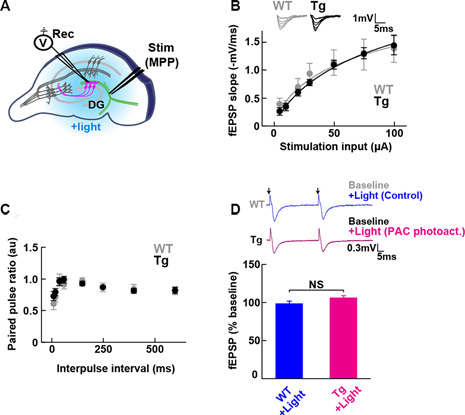
Baseline transmission at medial perforant path to granule dentate gyrus (MPP-DG) synapses of Tg and WT mice. **(A)** Schematic of the DG recording (Rec) electrode to detect fEPSPs upon stimulation (stim) of the MPP of hippocampal brain slices. Light was delivered for photoactivation (blue light, 480 ± 15 nm) of PAC expressed within granule neurons (magenta). **(B)** Input/output relationship within MPP-DG synapses in the absence (WT) and presence of PAC (Tg; WT *n* = 7 slices/3 mice, Tg *n* = 16 slices/9 mice). Inset shows sample I/O responses superimposed. **(C)** Comparison of the paired-pulse ratio (PPR) calculated from the ratio of the second fEPSP slope to the first, at different interpulse intervals (WT *n* = 12 slices/4 mice, Tg *n* = 15 slices/7 mice). **(D)** Quantification of synaptic response with/without PAC photoactivation for 60 s during the baseline recording (Tg *n* = 11 slices/7 mice, WT *n* = 13 slices/3 mice). NS, *p* > 0.05 (unpaired *t*-test). Data are presented as mean ± SEM. Upper: sample paired-pulse traces show the fEPSP responses before (Baseline) and 1 min after (+Light) light stimulation. Black arrows indicate the time of the single pulses of stimulation.

**Figure 3:**
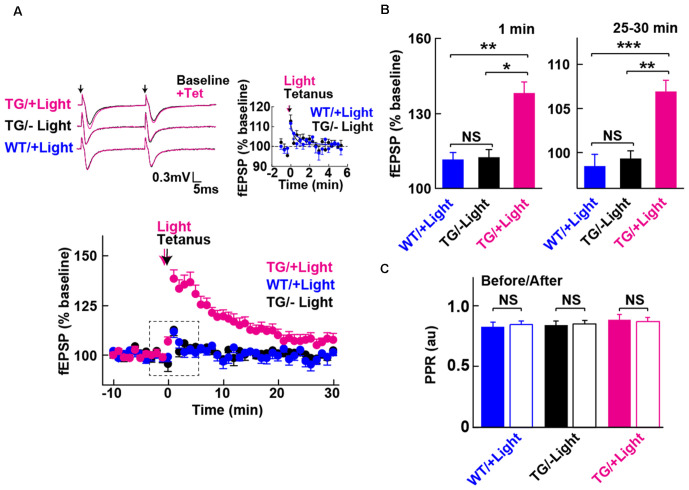
Long-term potentiation (LTP) enabled by photoactivation of PAC. **(A)** Lower: Blue light (480 nm, 5 min) photoactivation of PAC during tetanic stimulation (100 Hz × 4 trains, arrow) of MPP-DG synapses evoked potentiation of responses that persisted for 30 min. Hippocampal slices from WT mice receiving light, as well as Tg slices not receiving light, showed a transient and relatively small potentiation lasting about a minute (−Light, TG *n* = 10 slices/3 mice, +Light, TG *n* = 11 slices/7 mice, +Light, WT *n* = 13 slices/3 mice). Left upper: sample paired-pulse traces (scale bar of 0.3 mV and 5 ms) show superimposition of the fEPSP response before (Baseline; black) and 1 min after (+Tet; magenta) tetanic stimulation. Black arrows indicate the time of single pulses of stimulation. Right upper: comparison of post-tetanic potentiation within the boxed region in the lower graph showing the fEPSP normalized slope in expanded detail for the two control conditions, WT littermates in the presence of blue light (cyan) and Tg animals without blue light (black). **(B)** Quantification of synaptic potentiation at 1 min and 25–30 min after tetanus **(A)**. *p < 0.05, **p < 0.01, ***p < 0.001 (Tukey test). **(C)** Comparison of PPR before (−10 to 0 min) and after the tetanic stimulation (10–20 min) in the three conditions revealed no statistically significant differences (sample size identical to A). NS, *p* > 0.05 (paired *t*-test). All data displayed are mean ± SEM.

**Figure 4:**
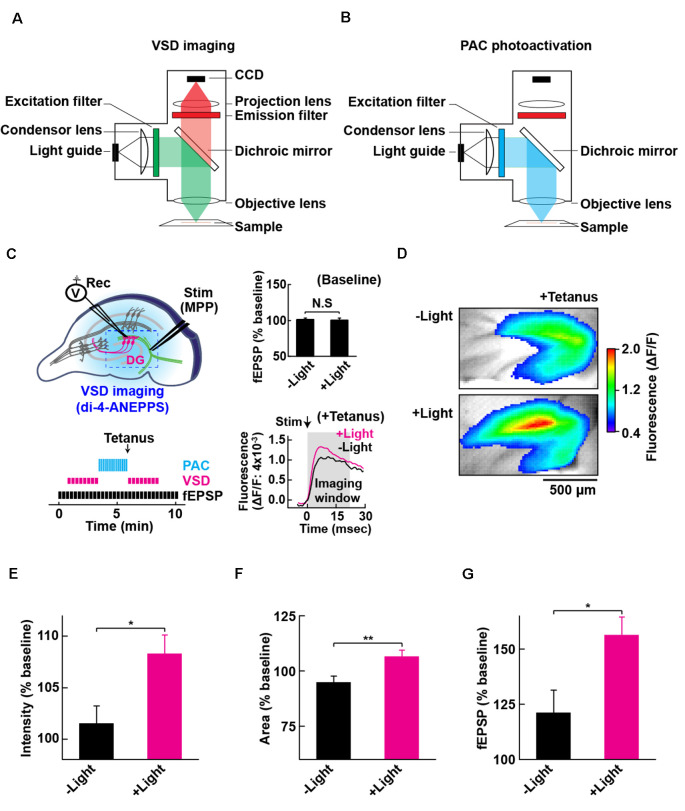
Configuration for PAC photoactivation and voltage-sensitive dye (VSD) imaging. A schematic of the setup for VSD imaging **(A)** and PAC photoactivation **(B)** in the fluorescence microscope. After PAC photoactivation, we switched the filter cube to detect VSD optical signal by the CCD-based camera system. The excitation light (530 ± 10 nm) for VSD imaging does not photoactivate PAC (down to 500 nm). **(C)** Left upper: schematic of PAC photoactivation, fEPSP recording and VSD imaging in PAC Tg hippocampal slices. The dotted rectangle indicates an ROI region for VSD imaging of fluorescence signal (di-4-ANEPPS). Left lower: time-course of VSD imaging with fEPSP recording before and after photoactivation of PAC with tetanic stimulation. VSD fluorescence changes were recorded as an average of eight images (400 ms duration each) taken every 20 s before and after tetanic stimulation (1 train, 100 × 100 Hz, 0.05 ms duration pulses). Right upper: quantification of synaptic response before/after PAC photoactivation for 60 s during the recording of VSD imaging (*n* = 11 slices/3 mice. NS, *p* > 0.05 (paired *t*-test). Data are presented as mean ± SEM. Right lower: trace of VSD fluorescence signal with/without PAC photoactivation (+Light/−Light). The black arrow (Stim) shows the time of delivery of tetanic stimulation which evoked the optical signals (at 0 ms). Gray box (Imaging analysis window): time window (0–20 ms) used for the measurement of fluorescence changes during basal stimulation and after the tetanic stimulation. **(D)** Representative VSD images in the DG area after tetanus with (+Light) or without (−Light) PAC photoactivation. The VSD fluorescence signal changes are displayed in pseudo color at the peak change time (6.4 ms). Warmer red colors indicate higher fluorescence intensities, signifying stronger depolarization. **(E–G)** Quantification of the changes **(C,D)** in the averaged peak fluorescence intensity **(E)** and area **(F)** during 0.4–160 s after tetanus (8 × 20 ms imaging every 20 s), and the fEPSP changes 0.05 ms after tetanus **(G)** with (+Light, *n* = 8 slices/2 mice) or without (−Light, *n* = 7 slices/2 mice) PAC photoactivation. *p < 0.05; **p < 0.01 (unpaired *t*-test). Data are presented as mean ± SEM.

**Figure 5:**
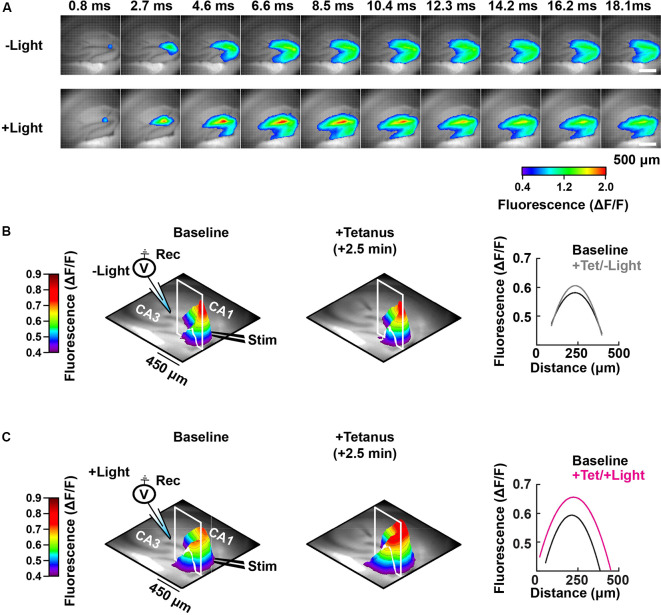
Spatial-temporal mapping of the PAC-induced enhancement of DG depolarization. **(A)** Representative time-lapse images of the VSD fluorescence change (depicted in pseudocolor scale) after tetanus, collected either without (−Light; upper row) or with photostimulation to activate PAC (+Light; lower row). **(B)** Left and middle: pseudo-colored 3D graph of the averaged peak VSD fluorescence change in the hippocampal DG region before (Baseline) and after tetanic stimulation (+Tetanus). The 3D fluorescence images were calculated as the ratio of the fluorescence changes before and after tetanus. Right: fluorescence profiles across the lines in the 3D fluorescence distribution (white line of left and middle) fitted with a parabola. Black, before tetanic stimulation (Baseline); grey, after tetanic stimulation without photostimulation to activate PAC (+Tet/−Light); magenta, after tetanic stimulation with the photostimulation (+Tet/+Light). The same analysis shown in panel **(B)** which was performed without PAC activation (−Light; *n* = 56 images/7 slices/2 mice), was repeated in panel **(C)** with PAC (+Light; *n* = 64 images/8 slices/2 mice) activation, respectively.

All recordings were made using a borosilicate pipette (5 μm inner diameter; filled with aCSF; ~1 MΩ). Responses were evoked and acquired every 20 s throughout the experiment using a Model 440 amplifier (Neurophase LLC, Palo Alto, CA, USA) digitized at 10 kHz (ITC-18; InstruTech Inc., NY, USA) and measured by the slope (10–50% of fEPSP rising phase). Tetanic stimulation was induced with a single train (100 Hz for 1 s × 0.05 ms duration pulses) without blocking inhibitory synapse function. Tetanic stimulation was induced immediately after PAC photostimulation. The fEPSP change was plotted by normalizing to the baseline slope (average of the 10-min period before tetanus).

### Statistical Analysis

Statistical methods are indicated in the figure legends. All data are presented as mean ± SEM. ***p < 0.001; **p < 0.01; *p < 0.05; NS, *p* > 0.05.

## Results

### Generation of a PAC Expressing Transgenic Mouse Line

To address the role of cAMP in synaptic plasticity at medial perforant path (MPP) fibers synapsing onto dentate gyrus (DG) granule neurons (MPP-DG synapses), we designed transgenic (Tg) mice expressing PAC. We constructed a transgene in which the CaMKIIα promoter drives the expression of a red fluorescence protein (RFP)-tagged PAC (Figure 1A). After generating several founders of Tg mice we genotyped them by PCR (Figure 1B), analyzed the expression pattern of RFP fluorescence in the brain and selected a mouse line which showed high expression of RFP-PAC in DG granule neurons, but not their input from the entorhinal cortex (Figure 1C). To validate the light-dependent enzymatic function of PAC, we photoactivated brain lysate from the hippocampal slices of Tg mice for 30 s (455 nm LED, 4.5 mW/mm^2^). The Tg lysates showed a light-dependent increase of cAMP compared with the brain lysate from the WT littermates, confirming that the expressed PAC actuator carries photoactivatable (cAMP generating) function within intact brain tissue (Figure 1D). These initial validation experiments on the Tg(CMV-Camk2a-RFP/PAC)3Koka new mouse line were carried out in the absence of any evoked axonal stimulation and set the stage for further studies on cAMP action on synaptic function *in situ*.

### Expressed PAC Does Not Affect Baseline MPP-DG Synaptic Transmission

To test whether the expression of PAC (without photoactivation) has any impact on the basal synaptic transmission at intact MPP-DG synapses, we measured the field excitatory postsynaptic potentials (fEPSPs) in the acutely prepared hippocampal slices (Figure 2A). The input/output (I/O) curves and paired-pulse ratio (PPR) showed no significant difference between PAC Tg animals and their WT littermates (Figures 2B,C), suggesting no effect of PAC expression on basal synaptic properties.

To test the effect of PAC photoactivation on basal synaptic responses at MPP-DG synapses, we next illuminated the slices with LED light (480 ± 15 nm, 1.5 mW for 60 s) under a fluorescence microscope and recorded the fEPSPs (Figure 2D). However, photoactivation of PAC alone did not affect basal synaptic transmission at MPP-DG synapses.

### Postsynaptic cAMP Enables Synaptic Potentiation

We next examined whether photoactivation of PAC has any effect on activity-dependent synaptic potentiation. The DG exhibits strong inhibition, such that tetanic stimulation of the MPP input is insufficient for generating LTP *in situ* unless GABA receptors are blocked (Wigstrom and Gustafsson, 1983; Nguyen and Kandel, 1996). However, upon photoactivation of PAC in combination with the tetanic stimulation of the MPP, we observed a robust synaptic potentiation that lasted for the entire 30 min of the post-tetanic recording period (Figures 3A,B, +Light, Tg). In contrast, in the absence of light delivery, tetanic stimulation of the MPP-DG synapses induced a weak and transient synaptic potentiation lasting only a few minutes (Figures 3A,B, −Light, Tg). Similarly, the WT littermates did not show the enhancement even with the same amount of light delivery upon tetanus (Figures 3A,B, +Light, WT). The PPR yields a short-term depression at MPP-DG synapses, a measure of the probability of release and short-term presynaptic plasticity. This parameter was not significantly altered 30 min after delivery of a tetanus either in the absence or presence of PAC photoactivation (Figure 3C). The data are consistent with an entirely postsynaptic effect, whereby tetanic potentiation in the presence of elevated cAMP leads to persistent potentiation of synaptic transmission.

### Combination of Optogenetic cAMP Manipulation With VSD Imaging

To evaluate the effect of cAMP on the global depolarization associated with the tetanus-induced potentiation, we employed fast (VSD: di-4-ANEPPS; Fluhler et al., 1985; Loew et al., 1992) imaging in combination with PAC photoactivation. For the optical recordings of membrane potential changes, we set up the custom-epifluorescence optical system equipped with a CMOS camera and a LED equipped with a feedback-stabilizing controller for constant LED light output (Figure 4A). In this system, the excitation wavelength (530 ± 10 nm) for VSD imaging has no overlap with the photoactivation wavelength of PAC (which required wavelengths up to 500 nm light). The excitation light was minimized for capturing the sufficient amount of VSD (di-4-ANEPPS) fluorescence needed for live high-speed imaging. For the photoactivation of PAC on the slices of PAC Tg mice, we switched the filter before blue light illumination (482 ± 17.5 nm; Figure 4B). Using VSD imaging, we optically monitored the depolarization that spread throughout the DG and also collected fEPSPs by electrophysiology (Figure 4C). Since the photoactivation of PAC alone did not affect basal synaptic transmission at MPP-DG synapses (Figure 4C right upper inset), we optically monitored the light-dependent cAMP effect on tetanus-induced fEPSP potentiation (Figure 4C right lower inset, 4D).

### Effect of cAMP on Depolarization During MPP-DG Potentiation

Photoactivation of PAC followed by tetanic stimulation showed an increased fluorescence intensity change in the DG area compared with controls receiving no 482 nm light (Figure 4E). The enhancement in depolarization occurred together with the fEPSP potentiation suggesting they are both consequences of cAMP generation (Figure 4G). In the absence of PAC photoactivation, the area of VSD fluorescence measured before vs. after the tetanus did not change (Figures 4F, 5, −Light). However, the region of fluorescence change was significantly enlarged by a tetanus that was preceded with photoactivation of PAC (Figures 4F, 5, +Light). A three-dimensional (3D) map of the averaged fluorescence changes visually shows the overall increase of both intensity and area of activity in the DG after tetanic stimulation of the MPP pathway in the presence of cAMP (photoactivation of PAC) compared with the tetanus alone (Figure 5). These results demonstrate that cAMP enhances the extent of dentate neuronal activation, enabling depolarization signals to propagate further.

## Discussion

In this study, we generated Tg mice overexpressing PAC in the granule neurons of the hippocampal DG. We prepared brain slices from the Tg mice and imaged the CaMKIIα-driven expression of PAC by way of the built-in RFP reporter. Confocal imaging revealed that the Tg(CMV-Camk2a-RFP/PAC)3Koka transgenic mice expressed abundant levels of RFP within the dentate granule cells, although further characterization is necessary to confirm the full specificity of the expression. This new mouse line did not show detectable levels of RFP expression in the DG input from the entorhinal cortex, suggesting exclusive and/or selective postsynaptic expression in the dentate granule neurons. Furthermore, the lysates from Tg slices showed photoactivity-dependent cAMP production upon delivery of blue light. In addition, tetanic activation of MPP to dentate synapses enabled LTP in Tg slices that received light during the tetanus. Presynaptic function was unaffected, indicating that when postsynaptic cAMP is elevated during high-frequency synaptic activity, there is a robust postsynaptic potentiation of synaptic strength that persists for at least 30 min.

### Optogenetic Manipulation of cAMP in Neurons

In addition to actuators such as PAC, other light-dependent cAMP production methods have also been reported. Opto-β2AR and its derivatives are chimeric rhodopsins that light-dependently release a G-protein subunit to activate adenylyl cyclase, AC (Airan et al., 2009). Compared to PAC, which directly produces AC upon light activation, this approach is indirect and acts through G-protein signaling pathways, that may conceivably affect multiple and diverse signaling cascades. Membrane-permeant caged cAMP is another method to instantaneously release cAMP by light (Moutinho et al., 2001; Nicol et al., 2007). However, this method is not ideal for repeated application over time, and a UV-requirement for uncaging prevents tissue penetrance and spatial specificity in tissues. Our results present an effective and consistent means to control and study the role of cAMP in native tissue.

### cAMP Elevation Results in the Spread of Depolarization in Granule Cells

To spatially investigate the amount and extent of granule cell activation by postsynaptic cAMP, we utilized (VSD) imaging to record changes in global DG depolarization upon MPP stimulation in acutely prepared hippocampal slices. We applied Di-4-ANNEPS for VSD imaging in combination with photostimulation of the PAC Tg brain slices. Di-4-ANNEPS is highly soluble in lipid solutions, remains longer in the membrane, making it suitable for long-term recordings of neural activity in brain slices (Tominaga et al., 2000, 2009, 2018). The excitation wavelength (530 ± 10 nm) for VSD imaging avoids photoactivation of PAC (<500 nm). The combination of optogenetic approaches to manipulate cAMP levels and image with VSD revealed that postsynaptic cAMP serves as a rapid positive modulator of excitation at MPP-DG synapses in hippocampal slices. Furthermore, cAMP expanded the activation area in the DG further along the MPP after tetanic stimulation. The enlarged region contains the soma and dendrites of granule cells, suggesting an increased number of activated synapses on the dendrites. The enlarged DG region of fluorescence change following tetanic stimulation paired with the optically-triggered generation of cAMP may indicate an increased number of activated synapses. For instance, cAMP may enhance the activity of weakly stimulated synapses (subthreshold stimulation for activation), and/or spread to more synapses to trigger heterosynaptic facilitation of LTP (Park et al., 2019). In principle, this photostimulation method for manipulating cAMP levels will be useful for characterizing the spatial-temporal features of this phenomenon.

### cAMP Elevation Enables LTP in Granule Cells Under Standard Slice Recording Conditions

Within brain slices, the strong DG inhibition normally precludes LTP to be induced by tetanic stimulation, unless GABA receptors are blocked (Wigstrom and Gustafsson, 1983; Nguyen and Kandel, 1996). It is therefore of interest that LTP could be readily induced without the need to pharmacologically remove synaptic inhibition when the tetanus was paired with cAMP elevation. Thus, cAMP circumvents the inhibitory influence of GABA-mediated synaptic inhibition. This property may be utilized by neuromodulatory systems, such as dopaminergic and noradrenergic, which can elevate cAMP within the dentate gyrus and facilitate LTP (Hamilton et al., 2010; Yang and Dani, 2014; Palacios-Filardo and Mellor, 2019). In this manner, powerful inhibitory processes restrict LTP until appropriate neuromodulatory inputs provide the necessary salience.

A potential mechanism for how cAMP reduces the influence of GABAergic inhibition relates to the spread and enhancement of depolarization of granule cells that we observed. Ordinarily, the synaptic activation of NMDA receptors is limited by the synaptic activation of GABA inhibition, which hyperpolarizes the membrane to intensify the Mg^2+^ block of NMDA receptors (Herron et al., 1985; Dingledine et al., 1986). The increase in depolarization would counteract this effect. Indeed, there was a trend towards a larger depolarizing field potential recorded immediately after the tetanic trains (PAC + light group vs. controls), which is consistent with enhanced synaptic activation of NMDA receptors. However, further experiments on current-clamped granule neurons are necessary to establish whether this is indeed the case. A candidate mechanism for this depolarization that we observed is the cyclic nucleotide-gated and -regulated channels, such as HCN1, that can be dynamically and rapidly modulated at the neuronal cell membrane (Robinson and Siegelbaum, 2003; Noam et al., 2010). An additional mechanism may involve the direct phosphorylation of NMDA receptors by PKA, following cAMP elevation (Skeberdis et al., 2006). Further experiments will need to be designed to examine these potential mechanisms.

An interesting feature of the cAMP-enabled LTP that we have described here is its rapid time-course of activation. Originally, cAMP was implicated in late-phase LTP, defined as the protein synthesis-dependent form of LTP that takes many tens of minutes to hours to develop (Frey et al., 1993; Matthies and Reymann, 1993; Huang and Kandel, 1994; Park et al., 2016). However, depending on the induction pattern, it became evident that cAMP-, AC-, and PKA are involved in early effects immediately after tetanic stimulation (Huang et al., 1994; Blitzer et al., 1995; Otmakhova et al., 2000). Furthermore, the application of rolipram, which inhibits the breakdown of cAMP, can greatly augment tetanus-induced LTP from its onset (Barad et al., 1998; Navakkode et al., 2004; Park et al., 2016). Therefore, under a variety of conditions, cAMP elevation can rapidly facilitate or permit synaptic plasticity.

In summary, we have developed a Tg mouse line expressing the actuator PAC and RFP reporter in granule neurons within hippocampal DG. We employed an optical system for photoactivation of PAC and simultaneously optically imaged membrane potential changes using the fast VSD di-4-ANEPPS in combination with electrophysiology. The work revealed that elevating postsynaptic cAMP at the same time as delivering tetanic stimulation results in a larger depolarization that spreads further within the dentate gyrus. It also enables the induction of LTP under conditions where LTP is not normally observed. We conclude that postsynaptic cAMP serves as a powerful modulator of synaptic plasticity at medial perforant path synapses onto dentate gyrus granule cells.

## Data Availability Statement

All datasets generated for this study are included in the article.

## Ethics Statement

The animal study was reviewed and approved by the animal care committee at The Centre for Phenogenomics (TCP; Toronto, ON, Canada) and Tokushima-Bunri University (Japan).

## Author Contributions

HL constructed a transgene and genotyped the founder lines made at TCP. HL and TL selected, maintained and backcrossed the PAC transgenic mouse after confirming DG expression with JG. JG carried out the pilot LTP work in the DG, which was followed up by TL. AA and JG prepared the hippocampal slices for the pure electrophysiology by TL in the GC lab. TL and JR performed RFP imaging studies with JG. MZ supports JR work. TL performed the combined electrophysiological and VSD imaging experiments in the TT lab. KO, JG, and TT designed the experiments. KO conceived the study. KO, TL, JG, TT, and GC wrote the manuscript.

## Conflict of Interest

The authors declare that the research was conducted in the absence of any commercial or financial relationships that could be construed as a potential conflict of interest.

## References

[B1] Abel T. Nguyen P. V. Barad M. Deuel T. A. Kandel E. R. Bourtchouladze R. (1997). Genetic demonstration of a role for PKA in the late phase of LTP and in hippocampus-based long-term memory. Cell 88, 615–626. 10.1016/s0092-8674(00)81904-2 9054501

[B2] Airan R. D. Thompson K. R. Fenno L. E. Bernstein H. Deisseroth K. (2009). Temporally precise *in vivo* control of intracellular signalling. Nature 458, 1025–1029. 10.1038/nature07926 19295515

[B3] Barad M. Bourtchouladze R. Winder D. G. Golan H. Kandel E. (1998). Rolipram, a type IV-specific phosphodiesterase inhibitor, facilitates the establishment of long-lasting long-term potentiation and improves memory. Proc. Natl. Acad. Sci. U S A 95, 15020–15025. 10.1073/pnas.95.25.15020 9844008 PMC24568

[B4] Blitzer R. D. Wong T. Nouranifar R. Iyengar R. Landau E. M. (1995). Postsynaptic cAMP pathway gates early LTP in hippocampal CA1 region. Neuron 15, 1403–1414. 10.1016/0896-6273(95)90018-7 8845163

[B5] Brandon E. P. Zhuo M. Huang Y. Y. Qi M. Gerhold K. A. Burton K. A.. (1995). Hippocampal long-term depression and depotentiation are defective in mice carrying a targeted disruption of the gene encoding the RI β-subunit of cAMP-dependent protein kinase. Proc. Natl. Acad. Sci. U S A 92, 8851–8855. 10.1073/pnas.92.19.8851 7568030 PMC41065

[B6] Chang P. Y. Jackson M. B. (2006). Heterogeneous spatial patterns of long-term potentiation in rat hippocampal slices. J. Physiol. 576, 427–443. 10.1113/jphysiol.2006.112128 16873414 PMC1890346

[B7] Collingridge G. L. Kehl S. J. McLennan H. (1983). The antagonism of amino acid-induced excitations of rat hippocampal CA1 neurones *in vitro*. J. Physiol. 334, 19–31. 10.1113/jphysiol.1983.sp014477 6134823 PMC1197297

[B8] Dingledine R. Hynes M. A. King G. L. (1986). Involvement of N-methyl-D-aspartate receptors in epileptiform bursting in the rat hippocampal slice. J. Physiol. 380, 175–189. 10.1113/jphysiol.1986.sp016279 2886653 PMC1182931

[B9] Fluhler E. Burnham V. G. Loew L. M. (1985). Spectra, membrane binding, and potentiometric responses of new charge shift probes. Biochemistry 24, 5749–5755. 10.1021/bi00342a010 4084490

[B10] Frey U. Huang Y. Y. Kandel E. R. (1993). Effects of camp simulate a late-stage of LTP in hippocampal Ca1 neurons. Science 260, 1661–1664. 10.1126/science.8389057 8389057

[B11] Gilbert P. E. Kesner R. P. Lee I. (2001). Dissociating hippocampal subregions: a double dissociation between dentate gyrus and CA1. Hippocampus 11, 626–636. 10.1002/hipo.1077 11811656

[B12] Govindarajan A. Israely I. Huang S.-Y. Tonegawa S. (2011). The dendritic branch is the preferred integrative unit for protein synthesis-dependent LTP. Neuron 69, 132–146. 10.1016/j.neuron.2010.12.008 21220104 PMC3032443

[B13] Grinvald A. Hildesheim R. (2004). VSDI: a new era in functional imaging of cortical dynamics. Nat. Rev. Neurosci. 5, 874–885. 10.1038/nrn1536 15496865

[B14] Hamilton T. J. Wheatley B. M. Sinclair D. B. Bachmann M. Larkum M. E. Colmers W. F. (2010). Dopamine modulates synaptic plasticity in dendrites of rat and human dentate granule cells. Proc. Natl. Acad. Sci. U S A 107, 18185–18190. 10.1073/pnas.1011558107 20921404 PMC2964233

[B15] Henderson J. T. Georgiou J. Jia Z. Robertson J. Elowe S. Roder J. C.. (2001). The receptor tyrosine kinase EphB2 regulates NMDA-dependent synaptic function. Neuron 32, 1041–1056. 10.1016/s0896-6273(01)00553-0 11754836

[B16] Herron C. E. Lester R. A. Coan E. J. Collingridge G. L. (1985). Intracellular demonstration of an N-methyl-D-aspartate receptor mediated component of synaptic transmission in the rat hippocampus. Neurosci. Lett. 60, 19–23. 10.1016/0304-3940(85)90375-1 2997672

[B17] Homma R. Baker B. J. Jin L. Garaschuk O. Konnerth A. Cohen L. B.. (2009). Wide-field and two-photon imaging of brain activity with voltage- and calcium-sensitive dyes. Methods Mol. Biol. 489, 43–79. 10.1007/978-1-59745-543-5_3 18839087

[B19] Huang Y. Y. Li X. C. Kandel E. R. (1994). cAMP contributes to mossy fiber LTP by initiating both a covalently mediated early phase and macromolecular synthesis-dependent late phase. Cell 79, 69–79. 10.1016/0092-8674(94)90401-4 7923379

[B18] Huang Y. Y. Kandel E. R. (1994). Recruitment of long-lasting and protein kinase A-dependent long-term potentiation in the CA1 region of hippocampus requires repeated tetanization. Learn. Mem. 1, 74–82. 10467587

[B20] Iseki M. Matsunaga S. Murakami A. Ohno K. Shiga K. Yoshida K.. (2002). A blue-light-activated adenylyl cyclase mediates photoavoidance in Euglena gracilis. Nature 415, 1047–1051. 10.1038/4151047a 11875575

[B21] Jansen V. Alvarez L. Balbach M. Strünker T. Hegemann P. Kaupp U. B.. (2015). Controlling fertilization and cAMP signaling in sperm by optogenetics. eLife 4:e05161. 10.7554/eLife.05161 25601414 PMC4298566

[B22] Kim K. Lakhanpal G. Lu H. E. Khan M. Suzuki A. Hayashi M. K.. (2015). A temporary gating of actin remodeling during synaptic plasticity consists of the interplay between the kinase and structural functions of CaMKII. Neuron 87, 813–826. 10.1016/j.neuron.2015.07.023 26291163 PMC4548268

[B23] Loew L. M. Cohen L. B. Dix J. Fluhler E. N. Montana V. Salama G.. (1992). A naphthyl analog of the aminostyryl pyridinium class of potentiometric membrane dyes shows consistent sensitivity in a variety of tissue, cell, and model membrane preparations. J. Membr. Biol. 130, 1–10. 10.1007/bf00233734 1469705

[B24] Matthies H. Reymann K. G. (1993). Protein kinase A inhibitors prevent the maintenance of hippocampal long-term potentiation. Neuroreport 4, 712–714. 10.1097/00001756-199306000-00028 8347813

[B25] Moser E. I. Krobert K. A. Moser M. B. Morris R. G. M. (1998). Impaired spatial learning after saturation of long-term potentiation. Science 281, 2038–2042. 10.1126/science.281.5385.2038 9748165

[B26] Moutinho A. Hussey P. J. Trewavas A. J. Malhó R. (2001). cAMP acts as a second messenger in pollen tube growth and reorientation. Proc. Natl. Acad. Sci. U S A 98, 10481–10486. 10.1073/pnas.171104598 11517303 PMC56986

[B27] Murakoshi H. Shin M. E. Parra-Bueno P. Szatmari E. M. Shibata A. C. E. Yasuda R. (2017). Kinetics of endogenous CaMKII required for synaptic plasticity revealed by optogenetic kinase inhibitor. Neuron 94, 37.e5–47.e5. 10.1016/j.neuron.2017.02.036 28318784 PMC5425291

[B28] Navakkode S. Sajikumar S. Frey J. U. (2004). The type IV-specific phosphodiesterase inhibitor rolipram and its effect on hippocampal long-term potentiation and synaptic tagging. J. Neurosci. 24, 7740–7744. 10.1523/JNEUROSCI.1796-04.2004 15342741 PMC6729613

[B29] Nguyen P. V. Kandel E. R. (1996). A macromolecular synthesis-dependent late phase of long-term potentiation requiring cAMP in the medial perforant pathway of rat hippocampal slices. J. Neurosci. 16, 3189–3198. 10.1523/JNEUROSCI.16-10-03189.1996 8627357 PMC6579127

[B30] Nicol X. Voyatzis S. Muzerelle A. Narboux-Nême N. Südhof T. C. Miles R.. (2007). cAMP oscillations and retinal activity are permissive for ephrin signaling during the establishment of the retinotopic map. Nat. Neurosci. 10, 340–347. 10.1038/nn1842 17259982

[B31] Noam Y. Zha Q. Phan L. Wu R. L. Chetkovich D. M. Wadman W. J.. (2010). Trafficking and surface expression of hyperpolarization-activated cyclic nucleotide-gated channels in hippocampal neurons. J. Biol. Chem. 285, 14724–14736. 10.1074/jbc.M109.070391 20215108 PMC2863223

[B32] Otmakhova N. A. Otmakhov N. Mortenson L. H. Lisman J. E. (2000). Inhibition of the cAMP pathway decreases early long-term potentiation at CA1 hippocampal synapses. J. Neurosci. 20, 4446–4451. 10.1523/JNEUROSCI.20-12-04446.2000 10844013 PMC6772463

[B33] Palacios-Filardo J. Mellor J. R. (2019). Neuromodulation of hippocampal long-term synaptic plasticity. Curr. Opin. Neurobiol. 54, 37–43. 10.1016/j.conb.2018.08.009 30212713 PMC6367596

[B34] Park P. Kang H. Sanderson T. M. Bortolotto Z. A. Georgiou J. Zhuo M.. (2018). The role of calcium-permeable AMPARs in long-term potentiation at principal neurons in the rodent hippocampus. Front. Synaptic Neurosci. 10:42. 10.3389/fnsyn.2018.00042 30524263 PMC6262052

[B35] Park P. Kang H. Sanderson T. M. Bortolotto Z. A. Georgiou J. Zhuo M.. (2019). On the role of calcium-permeable AMPARs in long-term potentiation and synaptic tagging in the rodent hippocampus. Front. Synaptic Neurosci. 11:4. 10.3389/fnsyn.2019.00004 30923499 PMC6426746

[B36] Park P. Sanderson T. M. Amici M. Choi S. L. Bortolotto Z. A. Zhuo M.. (2016). Calcium-permeable AMPA receptors mediate the induction of the protein kinase A-dependent component of long-term potentiation in the hippocampus. J. Neurosci. 36, 622–631. 10.1523/jneurosci.3625-15.2016 26758849 PMC4710778

[B37] Peterka D. S. Takahashi H. Yuste R. (2011). Imaging voltage in neurons. Neuron 69, 9–21. 10.1016/j.neuron.2010.12.010 21220095 PMC3387979

[B38] Robinson R. B. Siegelbaum S. A. (2003). Hyperpolarization-activated cation currents: from molecules to physiological function. Annu. Rev. Physiol. 65, 453–480. 10.1146/annurev.physiol.65.092101.142734 12471170

[B39] Ryu M.-H. Moskvin O. V. Siltberg-Liberles J. Gomelsky M. (2010). Natural and engineered photoactivated nucleotidyl cyclases for optogenetic applications. J. Biol. Chem. 285, 41501–41508. 10.1074/jbc.M110.177600 21030591 PMC3009876

[B40] Skeberdis V. A. Chevaleyre V. Lau C. G. Goldberg J. H. Pettit D. L. Suadicani S. O.. (2006). Protein kinase A regulates calcium permeability of NMDA receptors. Nat. Neurosci. 9, 501–510. 10.1038/nn1664 16531999

[B41] Stierl M. Stumpf P. Udwari D. Gueta R. Hagedorn R. Losi A.. (2011). Light modulation of cellular cAMP by a small bacterial photoactivated adenylyl cyclase, bPAC, of the soil bacterium Beggiatoa. J. Biol. Chem. 286, 1181–1188. 10.1074/jbc.M110.185496 21030594 PMC3020725

[B46] Tominaga Y. Ichikawa M. Tominaga T. (2009). Membrane potential response profiles of CA1 pyramidal cells probed with voltage-sensitive dye optical imaging in rat hippocampal slices reveal the impact of GABA_A_-mediated feed-forward inhibition in signal propagation. Neurosci. Res. 64, 152–161. 10.1016/j.neures.2009.02.007 19428695

[B42] Tominaga T. Kajiwara R. Tominaga Y. (2013). VSD imaging method of *ex vivo* brain preparation. J. Neurosci. Neuroeng. 2, 211–219. 10.1166/jnsne.2013.1051

[B47] Tominaga Y. Taketoshi M. Maeda N. Tominaga T. (2019). Wide-field single-photon optical recording in brain slices using voltage-sensitive dye. J. Vis. Exp. 148:e59692. 10.3791/59692 31282882

[B48] Tominaga Y. Taketoshi M. Tominaga T. (2018). Overall assay of neuronal signal propagation pattern with long-term potentiation (LTP) in hippocampal slices from the CA1 area with fast voltage-sensitive dye imaging. Front. Cell. Neurosci. 12:389. 10.3389/fncel.2018.00389 30405360 PMC6207578

[B43] Tominaga T. Tominaga Y. (2016). Paired burst stimulation causes GABA_A_ receptor-dependent spike firing facilitation in CA1 of rat hippocampal slices. Front. Cell. Neurosci. 10:9. 10.3389/fncel.2016.00009 26858604 PMC4731501

[B44] Tominaga T. Tominaga Y. Ichikawa M. (2002). Optical imaging of long-lasting depolarization on burst stimulation in area CA1 of rat hippocampal slices. J. Neurophysiol. 88, 1523–1532. 10.1152/jn.2002.88.3.1523 12205172

[B45] Tominaga T. Tominaga Y. Yamada H. Matsumoto G. Ichikawa M. (2000). Quantification of optical signals with electrophysiological signals in neural activities of Di-4-ANEPPS stained rat hippocampal slices. J. Neurosci. Methods 102, 11–23. 10.1016/s0165-0270(00)00270-3 11000407

[B49] Wigstrom H. Gustafsson B. (1983). Large long-lasting potentiation in the dentate gyrus *in vitro* during blockade of inhibition. Brain Res. 275, 153–158. 10.1016/0006-8993(83)90428-6 6313124

[B50] Wong S. T. Athos J. Figueroa X. A. Pineda V. V. Schaefer M. L. Chavkin C. C.. (1999). Calcium-stimulated adenylyl cyclase activity is critical for hippocampus-dependent long-term memory and late phase LTP. Neuron 23, 787–798. 10.1016/s0896-6273(01)80036-2 10482244

[B51] Yang K. Dani J. A. (2014). Dopamine D1 and D5 receptors modulate spike timing-dependent plasticity at medial perforant path to dentate granule cell synapses. J. Neurosci. 34, 15888–15897. 10.1523/JNEUROSCI.2400-14.2014 25429131 PMC4244463

[B52] Zhou Z. Tanaka K. F. Matsunaga S. Iseki M. Watanabe M. Matsuki N.. (2016). Photoactivated adenylyl cyclase (PAC) reveals novel mechanisms underlying cAMP-dependent axonal morphogenesis. Sci. Rep. 5:19679. 10.1038/srep19679 26795422 PMC4726437

